# A preliminary study of micro-RNAs as minimally invasive biomarkers for the diagnosis of prostate cancer patients

**DOI:** 10.1186/s13046-021-01875-0

**Published:** 2021-02-23

**Authors:** Simona Giglio, Cosimo De Nunzio, Roberto Cirombella, Antonella Stoppacciaro, Omar Faruq, Stefano Volinia, Gustavo Baldassarre, Andrea Tubaro, Hideshi Ishii, Carlo M. Croce, Andrea Vecchione

**Affiliations:** 1 University of Rome “Sapienza”, Via di Grottarossa 1035, 00198 Rome, Italy; 2 Department of morphological surgery and experimental medicine, Università degli Studi, Via Fossato di Mortara 64b, 44121 Ferrara, Italy; 3 Division of Molecular Oncology, CRO National Cancer Institute, Via Franco Gallini, 2, 33081 Aviano, Italy; 4 Osaka University Graduate School of Medicine, Center of Medical Innovation and Translational Research (CoMIT: 081), Suita, Yamadaoka 2-2, Osaka, 565-0871 Japan; 5 Department of Cancer Genetics, The Ohio University, 460W12th Ave, Columbus, OH 43210 USA

**Keywords:** microRNAs, Prostate cancer, Diagnosis, Prognosis

## Abstract

**Background:**

A prostate cancer diagnosis is based on biopsy sampling that is an invasive, expensive procedure, and doesn’t accurately represent multifocal disease.

**Methods:**

To establish a model using plasma miRs to distinguish Prostate cancer patients from non-cancer controls, we enrolled 600 patients histologically diagnosed as having or not prostate cancer at biopsy. Two hundred ninety patients were eligible for the analysis. Samples were randomly divided into discovery and validation cohorts.

**Results:**

NGS-miR-expression profiling revealed a miRs signature able to distinguish prostate cancer from non-cancer plasma samples. Of 51 miRs selected in the discovery cohort, we successfully validated 5 miRs (4732-3p, 98-5p, let-7a-5p, 26b-5p, and 21-5p) deregulated in prostate cancer samples compared to controls (*p* ≤ 0.05). Multivariate and ROC analyses show miR-26b-5p as a strong predictor of PCa, with an AUC of 0.89 (CI = 0.83–0.95;*p* < 0.001). Combining miRs 26b-5p and 98-5p, we developed a model that has the best predictive power in discriminating prostate cancer from non-cancer (AUC = 0.94; CI: 0,835-0,954).

To distinguish between low and high-grade prostate cancer, we found that miR-4732-3p levels were significantly higher; instead, miR-26b-5p and miR-98-5p levels were lower in low-grade compared to the high-grade group (*p* ≤ 0.05).

Combining miR-26b-5p and miR-4732-3p we have the highest diagnostic accuracy for high-grade prostate cancer patients, (AUC = 0.80; CI 0,69-0,873).

**Conclusions:**

Noninvasive diagnostic tests may reduce the number of unnecessary prostate biopsies.

The 2-miRs-diagnostic model (miR-26b-5p and miR-98-5p) and the 2-miRs-grade model (miR-26b-5p and miR-4732-3p) are promising minimally invasive tools in prostate cancer clinical management.

**Supplementary Information:**

The online version contains supplementary material available at 10.1186/s13046-021-01875-0.

## Background

Prostate cancer (PCa) is a leading cause of male cancer-related mortality in the United States, with an estimated of more than 175,000 diagnosed men and more than 32,000 deaths from the disease in 2019 [1]. PCa is a remarkably heterogeneous disease with tumors ranging from indolent to very aggressive [2].

A significant challenge in PCa clinical management is posed by the inability of the current diagnostic tests, such as serum Prostate-specific antigen (PSA) testing, digital rectal examination (DRE), to diagnose prostate cancer accurately and to discern between indolent and aggressive disease [3–6]. Although Magnetic Resonance Imaging has recently been demonstrated to identify PCa patients and particular clinically significant cancer accurately, it still presents concerns about health care systems resources to ensure quality, reproducibility, accessibility, cost-effectiveness, and adequate training [7, 8].

Owing to inherent limitations of serum PSA screening, including lack of specificity, this has led to PCa overdiagnosis and overtreatment [3, 5]. Therefore identification of aggressive versus indolent tumors and predicting PCa outcomes remain a significant clinical challenge [9, 10]. Several promising alternative tissue-based assays are being developed based on the molecular characterization of primary and metastatic prostate tumors that show improved sensitivity and specificity over PSA [4]. However, these assays are based on biopsy sampling, an invasive, expensive procedure, and does not accurately represent multifocal diseases.

Recent studies have demonstrated that aberrant expressions of miRs are closely associated with the development, invasion, metastasis, and prognosis of various cancers, including prostate cancer [11].

While most miRs are found at the intracellular level, many have been observed outside the cells, including in various body fluids [12, 13].

This study aimed to investigate the hypothesis that changes in circulating miRs represent potentially useful minimally invasive biomarkers for the diagnosis of prostate cancer.

## Methods

### Patients

Our analyses involved consecutive prospectively collected cohorts of patients followed-up in the Urology’s division at S. Andrea University-Hospital scheduled for prostate biopsy based on abnormal digital rectal examination or an elevated PSA. The enrollment period started in 2015 and ended in 2018. Twelve cores TRUS prostate biopsy was performed in all enrolled patients as described previously [14, 15]. Patients were randomly selected using closed envelops in three groups: Discovery cohort, Validation 1, and Validation 2.

We used a discovery cohort (37 PCa, 33 Benign prostate hyperplasia, BPH) to screen the plasma abundance of miRs, a validation cohort 1 (45 PCa, 45 BPH) to test results reproducibility, and then a validation cohort 2 (45 PCa, 65 BPH) to further test reproducibility in a consecutive randomized population. Samples were collected at the time of the biopsy.

**Fig. 1:**
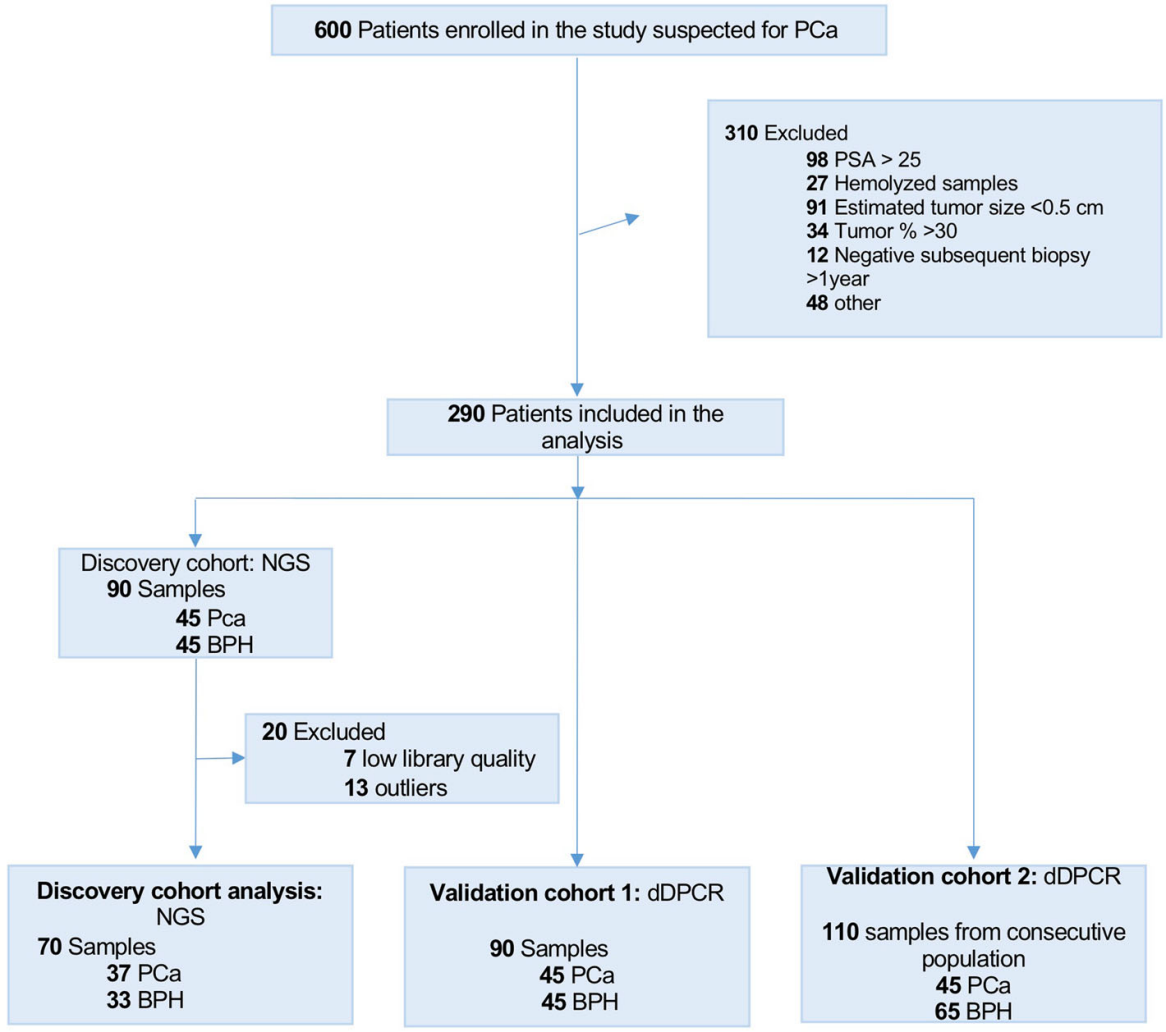
Flow diagram of discovery and validation approaches

The institutional ethics committee board authorized this study at S. Andrea University-Hospital Rome, Italy (Aut. #5176/2013). Written informed consent was obtained from all patients enrolled. The workflow of the discovery and validation approach is summarized in Fig. 1.

### RNA extraction and NGS

Briefly, aliquots of 200ul of plasma samples were thawed on ice, and 1 ml of QIazol Lysis Reagent (Qiagen) was added to the samples and incubated at room temperature for 5 min. Synthetic spike-in RNAs oligos were added to samples as a control for extraction and subsequent steps. RNA was eluted in 35 ul of nuclease-free water. Before use, RNA concentration in each sample was assayed with an ND-1000 spectrophotometer (NanoDrop). Its quality was assessed with the Agilent 2100 Bioanalyzer with Agilent RNA 6000 nano kit (Agilent Technologies, Santa Clara, CA, USA).

Indexed libraries were prepared from 1 μg/ul purified RNA with TruSeq Stranded Total RNA (Illumina) Library Prep Kit according to the manufacturer’s instructions. Libraries were quantified using the Agilent 2100 Bioanalyzer (Agilent Technologies) and pooled. Each index-tagged sample was present in equimolar amounts, with a final concentration of the pooled samples of 2bnM. The pooled samples underwent cluster generation and sequencing using an Illumina HiSeq 2500 System (Illumina) in a 2 × 100 paired-end (RNA-seq) format. Genomix4Life s.r.l performed NGS analysis. (Baronissi SA, Italy).

### Droplet digital PCR analysis

miRs analysis was performed by Droplet Digital PCR (BioRad) using miRCURY LNA™ miRNA PCR Assays according to the manufacturer’s instructions. Briefly, 1.5 ul RNA was reversely transcribed to cDNA with miRCURY LNA™ Universal RT microRNA PCR, cDNA synthesis kit II in a 10 ul reaction mixture, according to manufacturer’s protocol (Qiagen). cDNA was diluted 80X and 20 ul of the reaction mixture containing 8ul of the RT product, 12 ul of Digital PCR supermix™ (Bio-Rad), and 0.25 or 0.5 or 1 ul of miRcury LNA miRNA PCR Assays (Qiagen) and DEPC water were loaded into a plastic cartridge with 70ul of QX100 Droplet Generation oil and then placed into the QX100 Droplet Generator (Bio-Rad). The droplets generated from each sample were transferred to a 96-well PCR plate (Eppendorf, Germany). PCR amplification was carried on a T100 thermal cycler (Applied Biosystems) at 95 °C for 10 min, followed by 40 cycles of 95 °C for 30 s and 56 or 58 °C for 1 min, then one cycle of 98 °C for 10 min, ending at 4 °C. The plate was then loaded on Droplet Reader (Bio-Rad) and read automatically. A no template control (no cDNA in PCR) and negative control for each reverse transcription reaction (RT-neg) were included in every assay run. Absolute quantification of each miR was calculated from the number of positive counts per panel using the Poisson distribution. The quantification of the target miRNAs was presented as the number of copies/ul of reaction. Raw sequence files (fastq files) underwent a quality control analysis using FASTQC (http://www.bioinformatics.babraham.ac.uk/projects/fastqc/). Small RNA-Seq data were analyzed using iSmaRT [16] with standard parameters, using miRBase v21 as a reference track. Only miR showing a raw expression value > 50 raw-reads in at least one sample were considered for further analysis. Differentially expressed miRs were identified using DESeq2 [17]. A cutoff of | FC | ≥ 2 and FDR ≤ 0.001 was used to select differentially expressed miR between PCa vs. BPH samples. Raw data are available in GEO with accession number GSE118038.

### Statistical analysis

Differences in miRs levels between sample groups were assessed using the Mann Whitney test. For comparison of more than 2 groups, one-way ANOVA or Kruskal-Wallis tests were used as appropriate. Pearson 흌2 test was used to compare qualitative variables represented as frequencies.

Each miR’s potential to distinguish prostate cancer groups and controls was investigated using ddPCR values in a Receiver Operating Characteristic (ROC) curve analysis.

Using multiple logistic regression with the enter method, the statistical significant variables as assessed in univariate analysis were entered and investigated as predictors of prostate cancer presence vs. absence and in a separate model comparing Gleason Grade Group 3 (Gleason score ≥ 4 + 3) vs. Gleason Grade Group 1–2 (Gleason score ≤ 3 + 4) among men with a cancer diagnosis at biopsy. The predictive miRs-based model’s performance characteristics were assessed by calibration plots. The x-axis represents the predicted probability, and the y-axis represents the observed accuracy of the biopsy. Decision curves were generated to compare the net benefit of the miRs-based test. Decision curves enable the reader to understand graphically if the miRs-based test is beneficial for diagnosing PCa or a high-grade PCa. A range of probabilities is beneficial and gives us a quantification of the benefit. Data are presented as median with interquartile range and mean ± standard deviation. Odds ratios and 95% CI were calculated for the parameters in each group using PCa negative and Gleason 3 + 4 as the reference group.

Statistically significant *P* values were set at *p < 0.05, **p < 0.001, NS non-significant. All tests were two-sided. The statistical analysis was performed using GraphPad Prism5 or SPSS statistical software.

## Results

### Cohort description and study design

The clinicopathological characteristics of training, validation1, and validation2 cohorts are described in Table 1 and Fig. 1.

**Table 1:** Clinical characteristics of patients (PCa) and controls (BPH) in the Training, Validation1 and Validation 2 cohorts

	Training cohort					Validation cohort 1						Validation cohort 2					
	All *n* = 70	BPH *n* = 37 52%	PCa *n* = 33 48%		p		All *n* = 90	BPH *n* = 45 50%	PCa *n* = 45 50%		***p***		All *n* = 110	BPH *n* = 65 59%	PCa n = 45 41%		p
Age (Years)	67.15 ± 9	65.2 ± 10	69.1 ± 8		.314	Age (Years)	68 ± 8	66 ± 8	69.8 ± 7.8		.061	Age (Years)	69 ± 7.3	67.1 ± 7	70 ± 7.2		.154
PSA (ng/ml)	7.8 ± 5.5	5.6 ± 2	10 ± 5		**.001**	PSA (ng/ml)	8.78 ± 6	8.7 ± 4.8	8.8 ± 5.6		.769	PSA (ng/ml)	7 ± 5.8	7 ± 5.3	7.2 ± 6.7		.874
TRUS Volume (ml)	53.5 ± 29	68 ± 28	39 ± 12		**.001**	TRUS Volume (ml)	51.8 ± 28	55 ± 23	48 ± 21		.321	TRUS Volume (ml)	55 ± 27	61	45.5 ± 19		.053
			**EG < 2** *n* = 14 42%	**EG > 3** *n* = 19 45%					**EG < 2** *n* = 12 27%	**EG > 3** n = 19 73%					**EG < 2** *n* = 28 62%	**EG > 3** *n* = 17 38%	
Age (Years)			69.8 ± 7.7	67 ± 8	.743	Age (Years)			68.5 ± 7.3	70 ± 8	.465	Age (Years)			69 ± 7	73 ± 7	.308
PSA (ng/ml)			10 ± 6.8	9.6 ± 4.5	.924	PSA (ng/ml)			5.8 ± 2	9.9 ± 6.2	**.022**	PSA (ng/ml)			6.5 ± 2.9	9.06 ± 7	**.029**
TRUS Volume (ml)			37 ± 12	40 ± 11	.487	TRUS Volume (ml)			48 ± 27	48 ± 18	.906	TRUS Volume (ml)			43.5 ± 19	54.5 ± 19	.664

*Abbreviations*: *BPH* Benign Prostate Hyperplasia, *PCa* Prostate cancer, *PSA* Prostate Specific Antigen, *TRUS* transrectal ultrasound, *EG* Epstein group. Data are presented as median ± standard deviation

Of the 600 patients enrolled in the study, 290 patients were suitable for the analysis (Fig. 1).

For the discovery cohort, we selected a total of 70 patients that underwent 12-core transrectal ultrasound (TRUS) biopsy, of which 48% had cancer at the diagnosis. The average of the clinical features was: age 67.15 (IR 55/75), PSA 7.8 ng/ml (IR 4.1/13.4), and the TRUS volume 53.5 ml (IR 29/86). Among all cancer patients, 58% showed a high-grade (Gleason score ≥ 4 + 3, Epstein group ≥III) cancer. Due to a bias in participants’ sampling, there was a significant difference in age and TRUS volume between prostatic cancer and control patients in the discovery cohort (Table 1).

For validation cohort1, we enrolled 90 patients that underwent 12-core TRUS biopsy, of which 50% had cancer at the diagnosis. The average of the clinical features was: age 68 (IR 61/75), PSA 8.78 ng/ml (IR 5.2/11.2), and the TRUS volume 51.8 ml (IR 35/67). Among all cancer patients, 73% showed a high-grade (Gleason score ≥ 4 + 3, Epstein group ≥III) cancer (Table 1).

For validation cohort 2, we enrolled a consecutive series of 110 patients that underwent 12-core TRUS biopsy, of which 41% had cancer at the diagnosis. The average of the clinical features was: age 69 (IR 44/80), PSA 7 ng/ml (IR5.12/11.7), and the TRUS volume 55 ml (IR 35/74). Unlike the training and cohort 1 set, only 30% of patients in the validation cohort 2 showed high-grade (Gleason score ≥ 4 + 3, Epstein group ≥III) cancer, reflecting the trend seen in an unselected patient population (Table 1).

### NGS analysis of circulating miRNAs in PCa

In the discovery phase, we aimed to explore the potential of plasma miRs as a diagnostic tool for PCa exploiting an NGS high-throughput analysis using Illumina HiSeq 2500 sequencing system.

The analysis revealed that plasma-derived from PCa patients showed a significant alteration in miRs expression compared to controls (Fig. S1 in the Supplement).

PCa patients displayed overexpression of 34 and downregulation of 17 miRs compared to BPH samples (Fold Change ≥2, FDR ≤0.001) (Fig. S1, Table S1). Cluster analysis identified two main clusters (C1 and 2) described in Table S1 in the Supplement.

### Validation of candidate miRNAs by droplet digital PCR

For validation, all miRs discovered in the NGS analysis with Fold Change ≥2 and FDR ≤0.001, were analyzed in validation cohort1 (Table 1), using droplet digital PCR (dDPCR).

**Fig. 2:**
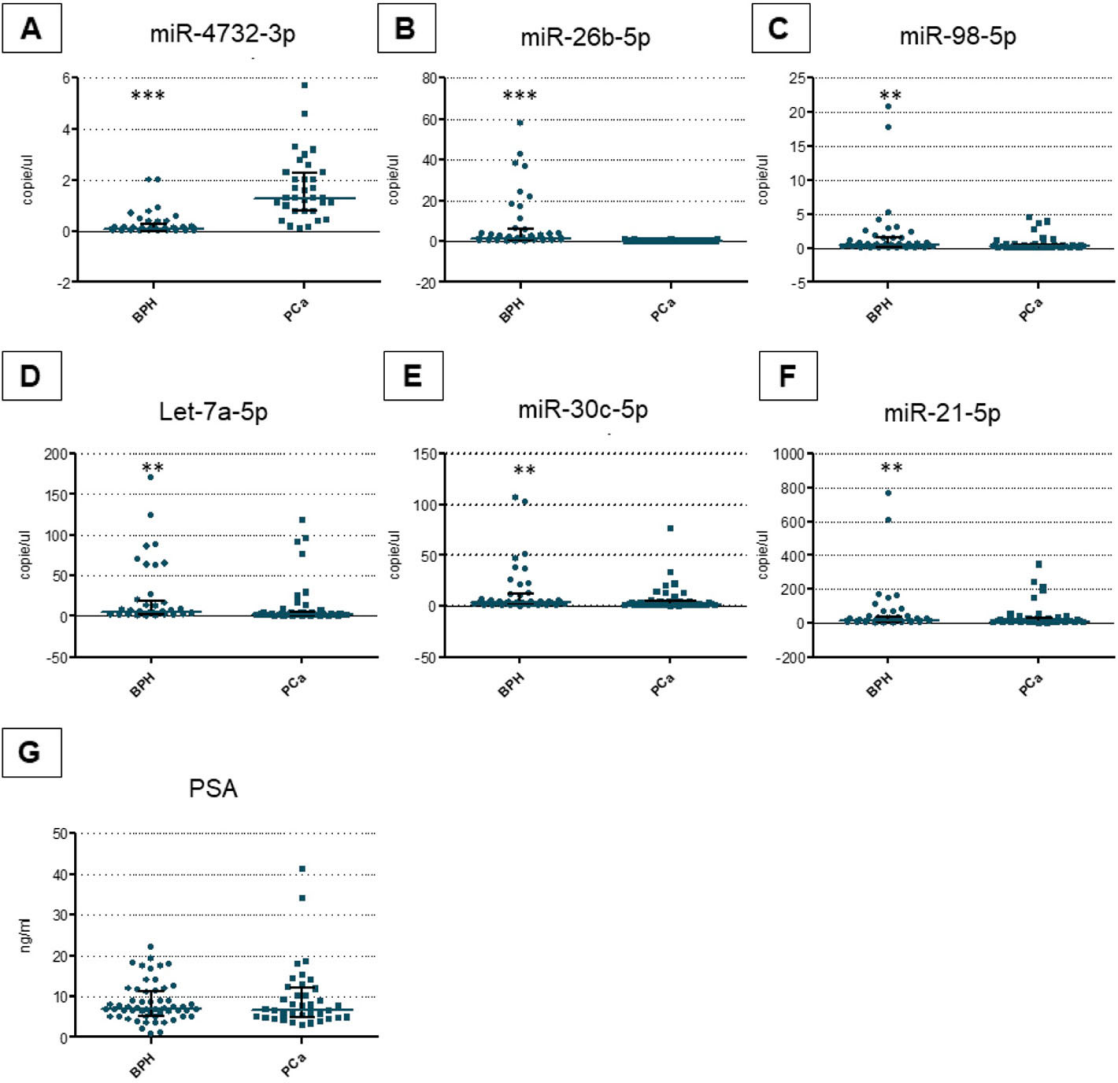
Differential levels of miRs and PSA in PCa patients versus controls in plasma samples from validation cohort 1. Abbreviations: PCA,Prostate Cancer; BPH,Benign prostate Hyperplasia; PSA,Prostate Specific Antigen. All data were expressed as copies/ μL of reaction or ng/ml as described. The horizontal blue line in the middle of each box indicates the median.Whereas the top and bottom black line 75th and 25th percentiles, respectively, Mann-Witney U test; ** indicates *p* value < 0.001; *** indicates *p* value < 0.0001; for all comparisons (BPH versus PCa)

**Fig. 3:**
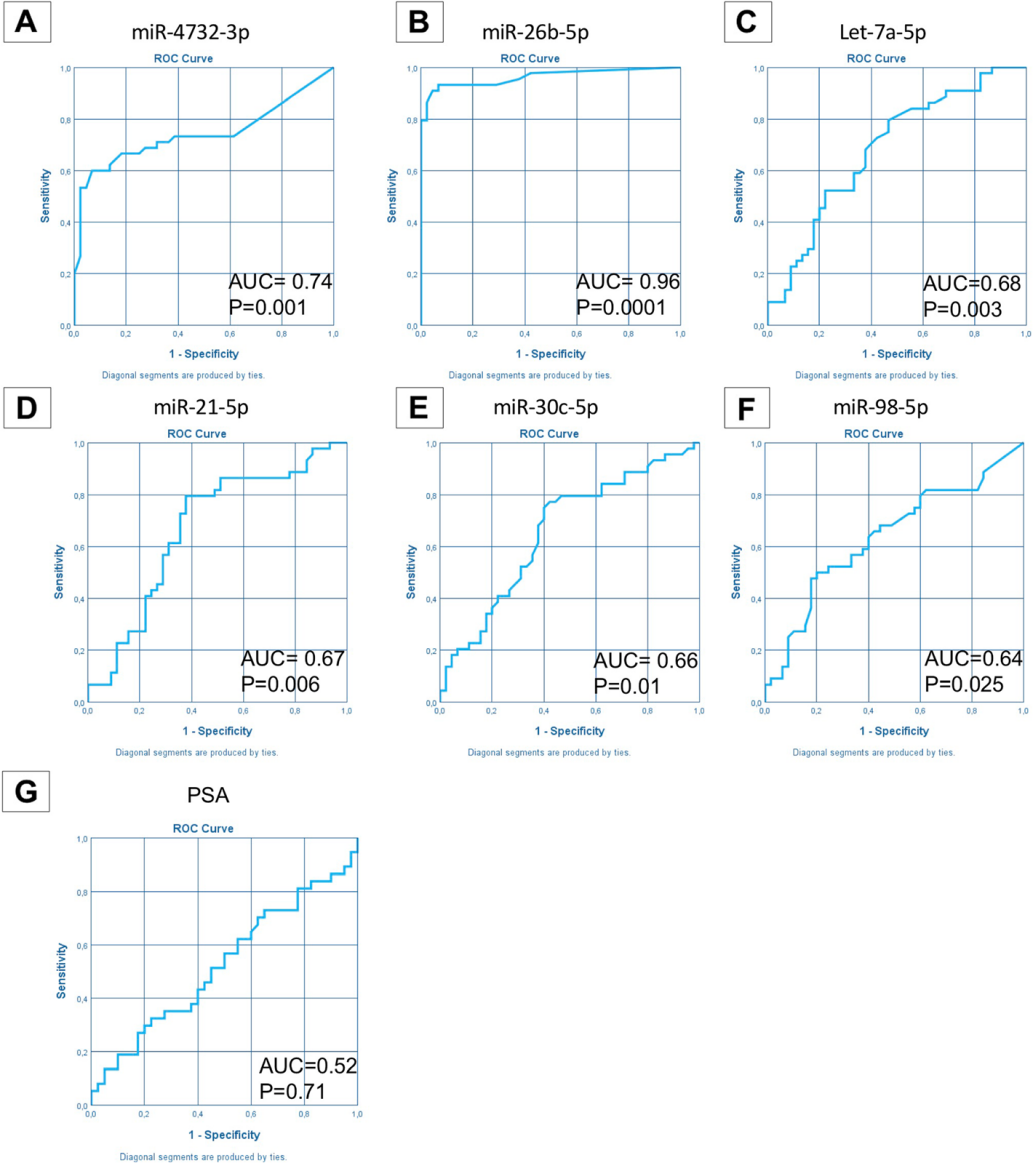
ROC curve analysis of selected miRs in plasma samples from validation cohort 1. Abbreviations: AUC, Area under the ROC Curve; *P*, *p* value

We successfully confirmed the downregulation in PCa samples of five miRs (miRs-98-5p, let-7a-5p, −26b-5p, −30c-5p and − 21-5p) and the upregulation of one miR (miR-4732-3p) compared to controls (*p*-value≤0.05). These results indicate that dysregulated miRs can discriminate between PCa and BPH (Figs. 2, 3 A-F). Importantly, no statistically significant differences in PSA concentration were observed between PCa and controls (Figs. 2, 3 G).

Logistic regression analysis demonstrated that miR-26b-5p is a strong, PSA independent predictor for PCa presence in univariate and multivariate analysis (univariate: *p* = 0.0001, CI 0.00–0.002; multivariate: *P* = 0.0001, CI 0.00–0.48) (Table 2 left panel).

**Table 2:** Univariate and Multivariate analysis according to cancer status in Validation1 and 2 cohorts

	Validation cohort 1				Validation cohort 2			
Variables	Univariate		Multivariate		Univariate		Multivariate	
	Odds ratio (CI)	p	Odds ratio (CI)	p	Odds ratio (CI)	p	Odds ratio (CI)	p
AGE	1.06(1.00–1.11)	**.036**	1.04(0.93–1.17)	.47	1.05(.99–1.11)	.68		
PSA	.97(0.87–1.07)	.54			1.05(1.00–1.11)	.52		
miR-26b-5p	.0001(.00–.002)	**.0001**	.0001(.00–.48)	**.0001**	.11(.001–.109)	**<.0001**	.011(.001–.11)	**.0001**
miR-30c	.98(0.95–1.01)	.14			.984(.96–1.101)	.27		
miR-21-5p	.99(0.99–1.00)	.21			.99(.99–1.00)	.29		
miR-98-5p	.77(0.54–1.10)	.160			.68(.43–1.09)	.11		
miR-4732-3p	6.60(2.46–17.37)	**.0001**	1.82(.55–6.04)	**.33**	1.53(1.01–2.33)	**.04**	.98(0.59–1.69)	.94
Let7a-5p	.99(.98–1.00)	.117			.99(.97–1.00)	.16		

*Abbreviations* CI, Confident Interval, *p p* value, *PSA* Prostate Specific Antigen. Data are presented as odds ratio (CI). Empty cells correspond to variables that were not measured. Baseline variables that achieved a level of significance of *p* < .05 in the univariate analysis were entered into multivariate models

**Fig. 4:**
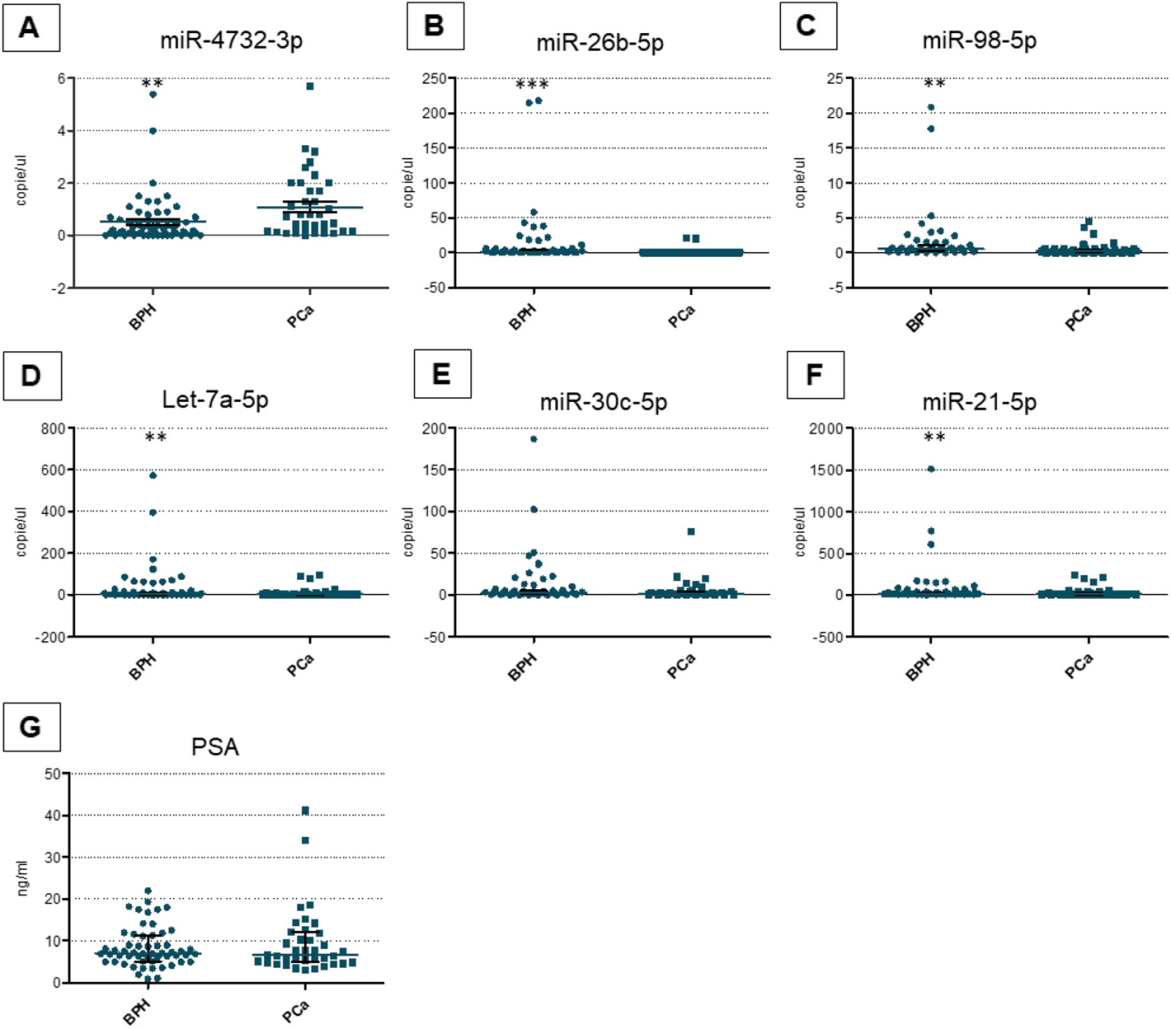
Differential levels of miRs and PSA in PCa patients versus controls (BPH) in plasma samples from validation cohort 2. Abbreviations: PCa, Prostate Cancer; BPH,Benign prostate Hyperplasia; PSA,Prostate Specific Antigen. All data were expressed as copies/ _μL_ of reaction or ng/ml as described. The horizontal blue line in the middle of each box indicates the median. Whereas the top and bottom black line 75th and 25th percentiles, respectively, Mann-Witney U test; ** indicates *p* value < 0.001; *** indicates *p* value < 0.0001; for all comparison (BPH versus PCa)

To better control the sampling bias, the robustness of our miRs signature’s predictive performance was assessed through validation cohort 2, derived from a consecutive population (Table 1). We confirmed the significant differences between tumors and controls of all miRs previously identified except for miR-30c. (Fig. 4). On multivariate analysis, miR-26b-5p resulted in an independent predictor of PCa (Table 2 right panel). Results from the different cohorts were coherent, supporting their consistency.

**Fig. 5:**
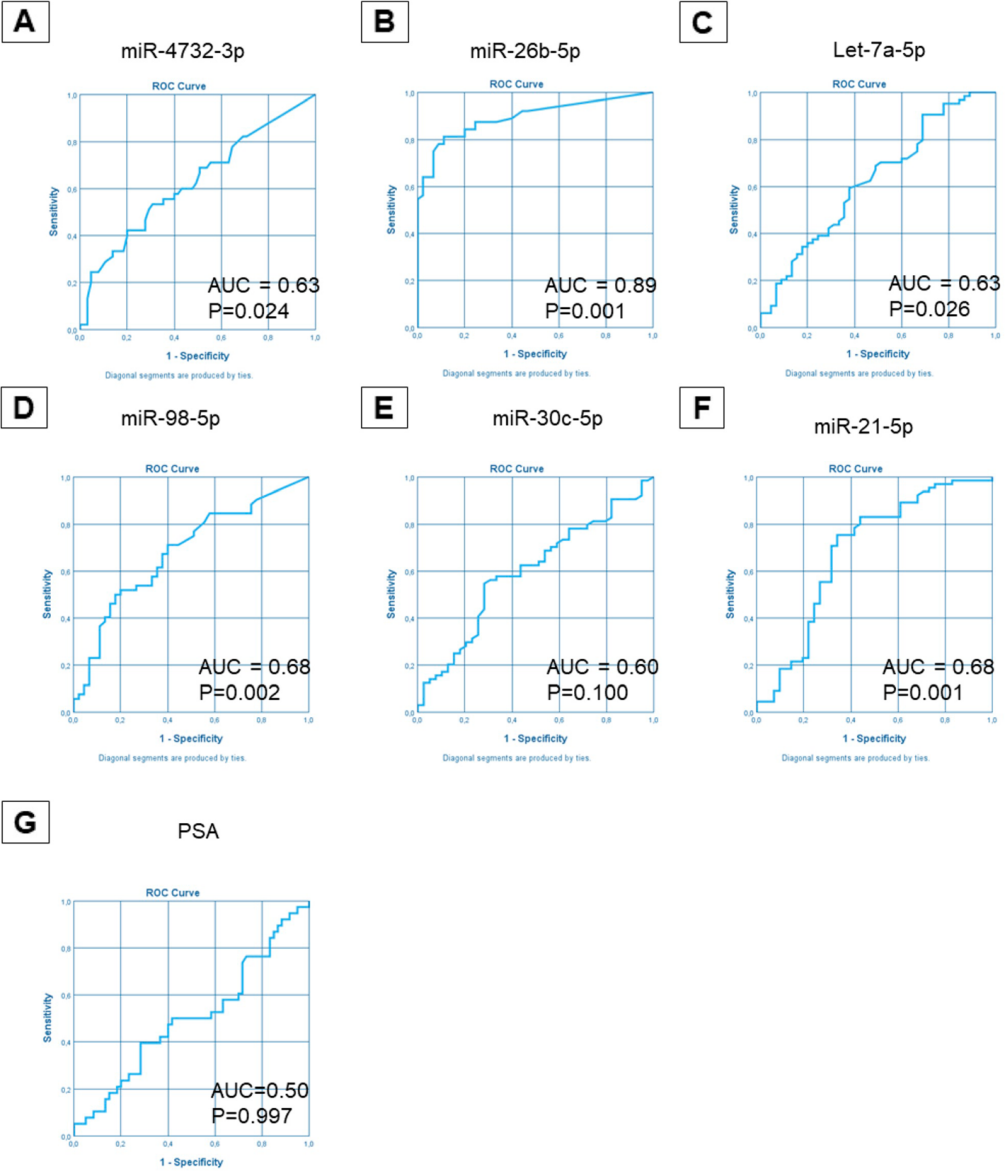
ROC curve analysis of selected miRs in plasma samples from validation cohort 2 according to cancer status. Abbreviations: AUC, Area under the ROC Curve; P, p value; PSA, Prostate Specific Antigen

ROC analysis for each identified miR confirmed the excellent diagnostic accuracies of miR-26b-5p in detecting PCa, with an AUC 0.89 (CI 0.83–0.95 *p* < 0.0001), and all miRs tested showed higher diagnostic power with respect to PSA (Fig. 5).

**Fig. 6:**
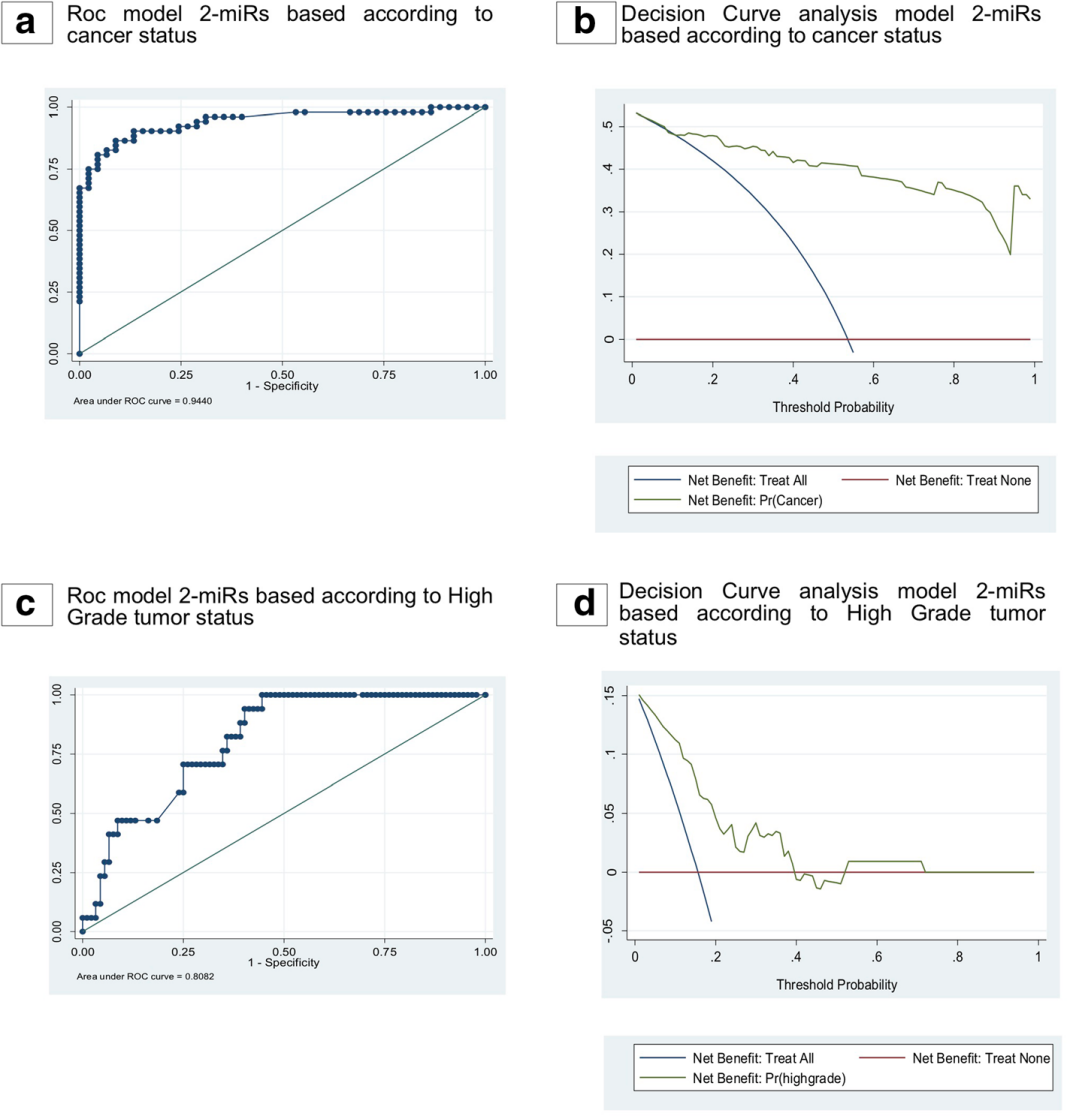
Decision Curve Analysis of the 2-miRs model in the Validation cohort 2. **a**. Roc model 2-miRs based according to cancer status in validation cohort 2. **b**. Decision curve analysis for the 2-miR model based according to cancer status in validation cohort 2. The straight red line at y = 0 represents a strategy of performing a biopsy in no patients and the blue line represents the strategy of performing a biopsy in all patietns. The area of the graph for which a risk calculator has a greater net benefit than both the ‘biopsy none’ and ‘biopsy all lines is where it has greatest clinical applicability. **c**. Roc model 2-miRs based according to High grade tumor status in validation cohort 2. **d**. Decision curve analysis for the 2-miRs model based according to grade tumor status in validation cohort 2. The straight red line at y = 0 represents a strategy of performing a biopsy in no patients and the blue line represents the strategy of performing a biopsy in all patietns. The area of the graph for which a risk calculator has a greater net benefit than both the ‘biopsy none’ and ‘biopsy all lines is where it has greatest clinical applicability

Based on the results from the validation cohort 2, the diagnostic performance of combining two or more miRs was assessed. A ROC model, including the combination of miR-26b-5p and miR-98-5p, showed the best predictive power in discriminating PCa from BPH patients, with an AUC of 0.944 (Fig. 6a).

Analyzing the calibration plots, our model demonstrates the agreement between observed and predicted probabilities for the diagnosis of PCa (Fig. S2A in Supplement).

The decision curve strongly supported that using our novel and noninvasive two-miRs based model represents the best strategy in diagnosing PCa. Remarkably, the model has a higher net benefit than both treat-all (blue line) and treat-non (red line) for the threshold probabilities between 0 and 90%. (Fig. 6b).

Given the different outcomes between low- and high-grade PCa, the possibility of identifying its grade is fundamental in PCa diagnosis and management.

To this aim, we evaluated the expression of our identified miRs in high-grade PCa (Gleason score ≥ 4 + 3, Epstein group ≥III) vs. No-Cancer+Low Grade Cancer (Gleason score ≤ 3 + 4, Epstein group ≤II).

Results showed that miR-4732-3p levels were significantly higher in the high-grade group compared to the No-Cancer+Low Grade group. In comparison, miR-26b-5p and miR-98-5p levels were considerably higher in the No-Cancer+Low Grade group than the high-grade group (Fig. S3 in Supplement). No statistically significant difference was found for PSA and miRs let7a-5p, −30c, and − 21-5p in the high-grade group compared to No-Cancer/Low-grade group.

Also, the multivariate logistic analysis showed that miR-26b-5p was an independent predictor of the presence of a high-grade PCa (Table 3).

**Table 3:** miRs prediction of patients with high Grade vs No-Cancer + Low Grade tumors in Validation cohort 2

	Validation cohort 2			
	High Grade vs No-Cancer + Low-grade			
Variables	**Univariate**		**Multivariate**	
	Odds ratio (CI)	p	Odds ratio (CI)	p
AGE	1.10(1.01–1.19)	**.027**	1.09(0.98–1.20)	.10
PSA	1.07(0.99–1.15)	.68		
miR-26b-5p	.015(.000–.60)	**.25**	.018(.001–.85)	**.041**
miR-30c	.97(.90–1.04)	.34		
miR-21-5p	.99(.98–1.01)	.34		
miR-98-5p	.62(0.27–1.44)	.62		
miR-4732-3p	1.65(1.09–2.50)	**.018**	1.4(.87–2.27)	.17
Let7a-5p	.99(.97–1.01)	.40		

*Abbreviations*: *CI* Confident Interval, *p p* value, *PSA* Prostate Specific Antigen. Data are presented as odds ratio (CI). Empty cells correspond to variables that were not measured. Baseline variables that achieved a level of significance of p < .05 in the univariate analysis were entered into multivariate models

ROC analysis showed that miR-26b-5p and miR-4732-3p had the highest diagnostic accuracies for high-grade prostate cancer patients with an AUC of 0.78 (CI 0.69–0.87; *p* = 0.0001) and 0.73 (CI 0.60–0.87; *p* = 0.002), respectively (Fig. S4).

A ROC model, including the combination of plasma miR-26b-5p and miR-4732-3p, showed the best predictive power in diagnosing high-grade PCa patients, displaying an AUC of 0.80 (Fig. 6c and Table S2 in Supplement). Analyzing the calibration plots, our model demonstrates the agreement between observed and predicted probabilities for the diagnosis of high-grade PCa (Fig. S5 in Supplement). The decision curve strongly supported that the use of our novel and noninvasive two-miRs based model represents the best strategy in the high-grade diagnosis of PCa (Fig. 6d). Notably, the model has a higher net benefit than both treat-all (blue line) and treat-non (red line) for the probabilities threshold between 0 and 40%. (Fig. 6d).

## Discussion

In PCa, diagnosis and tumor aggressiveness are some of the major challenges for its clinical management. Although PSA screening aided early detection and screening, its levels poorly correlate with tumor aggressiveness or dissemination, and they are not useful in predicting responsiveness or relapse.

Diagnostic accuracy, particularly in terms of risk stratification, initial staging, and active surveillance is one of the main issues in this field.

Circulating miRs profiles are a promising strategy for cancer diagnosis [18, 19], particularly in PCa, where minimally invasive, sensitive, and specific biomarkers are needed.

This study describes two novel panels of miRs derived from an in-depth analysis of patients’ plasma samples, one specific for diagnosing and one for grading PCa, respectively.

Previous investigations demonstrated the potential diagnostic value of circulating miRs as biomarkers for PCa [20–26]. The resulting miRs from this study were found to be modulated in different tumors.

Specifically, miR-98-5p was downregulated in lung cancer tissue compared to adjacent cancer-free tissue [27] and in the serum of lung cancer patients compared to controls [28].

Additionally, Wang et al. observed this downregulation was positively correlated with lymph node metastasis, worse TNM stage, and a decrease in patients’ overall survival. Similarly, miR-98-5p was reported to be downregulated in melanoma patients and metastatic melanoma compared to cancer-free controls [29].

MiR-98-5p was also observed to be downregulated in esophageal squamous cell carcinoma [30].

Some studies have also reported decreased serum *let*-*7a*, miR 26b-5p, 30c-5p levels in prostate cancer [31–33].

No data are available, to date, for miR-4732-3p.

The candidates’ miRs were selected in the discovery cohort in which we analyzed the entire MIRnome exploiting NGS technology. We first wanted to test the extremes (i.e., patients with known prostate cancer predominantly with high grade and stage and a tumor size > 2.5 cm vs. patients with known benign prostate hyperplasia with a negative biopsy result with at least 5 y of follow-up). This allowed us to identify a miRs signature that clearly distinguished the two conditions.

The first validation cohort confirmed a signature of 6 miRs with high predictive power in diagnosing PCa. Most importantly, each identified miR showed a predictive power superior and independent from PSA.

Since data driven from the first validation may tend not to reflect a real population (i.e., with a reasonable incidence of 30% of PCa and a 60% of high-grade tumors), we used a second validation cohort derived from a consecutive population.

Notably, dysregulated miRs were consistent through the second validation cohort, except for miR-30c that lacked statistical significance.

Based on these results, we first developed a test able to diagnose PCa. Among all the miRs identified in the analysis, we discovered and demonstrated that the combination of 2 miRs (mir-26b-5p and miR-98-5p) had the best predictive power (AUC 0.944) in diagnosing PCa.

Test performance was highlighted by the analysis of the decision curve, that showed a diagnostic accuracy that results in a higher net benefit in both groups treat-all and treat-non for the probabilities threshold between 0 and 90%, strongly supporting the use of our novel two-miRs based model as the best strategy in the diagnosis of PCa.

We developed a two miR-based test (miR-26b-5p and miR-4732-3p) to identify PCa grade (Low vs. High). A ROC model, including the combination of these two miRs, showed the best predictive power (AUC 0.80) in diagnosing high-grade PCa patients. Furthermore, this model’s performance assessment showed a higher net benefit than both treat-all and treat- non, for the probabilities threshold between 0 and 40%.

Our study’s strengths are the large number of miRs analyzed in the discovery cohort, the large selection of miRs candidates in two independent validation cohorts, and the use of dDPCR to confirm identified miRs.

Due to their low abundance in the plasma and the lack of an adequate normalization method, we have exploited the dDPCR technique’s ability to obtain absolute quantification of circulating miRs levels without the need for a reference gene. Furthermore, compared to real-time PCR, dDPCR shows lower variations; thus, it can better achieve statistically significant discrimination between tumors and controls. We must also acknowledge the important limitations of our study. This is a small single-center study with a limited number of patients affected by PCa and by high-grade cancer, which reduced a robust evaluation of the relationship between miRs and PCa grading in our series*.* The study results depend upon the enrolled population, and our patient’s characteristics may differ from what has been observed in other countries. However comparing the three cohorts of patients we found no significant differences (data not shown) in term of age, PSA value and prostate volume. Furthermore in all cohorts prostate cancer patients were significantly older and presented as expected smaller prostate volume confirming the validity of our cohorts [14, 15]. Our cohort characteristics describe an Italian pre-screening community, as shown by the high prevalence of high-grade PCa, which is undoubtedly representative of our geographical region. It may differ from racially diverse northern European, North American, South American, and Asian populations. Our cohort is entirely Caucasian, with no Africans and Hispanics. Our academic hospital operated under the Italian National Care System that never supported screening programs for prostate cancer.

Furthermore, it is assumable that our patients’ population had limited access to prostate cancer centers and screening programs in the past. Another possible limitation derives from using a biopsy cohort without confirmation from radical prostatectomy specimens or follow-up data and a pre-biopsy MRI recently recommended by the EAU guidelines. However, when we started our study in 2015, MRI was not considered a recommended test in the first set of prostate biopsies. So far, we have no data on pre-biopsy MRI lesion, PIRADS score, or prostate volume and local stage. To overcome some of these limitations, a multicenter study is ongoing evaluating the role of our miRs in patients with a PIRADS score three at MRI and in patients with locally advanced disease, as shown by MRI.

Notwithstanding all these limitations, in our study, we observed that NGS-miR-expression profiling revealed a miRs signature able to distinguish prostate cancer from non-cancer plasma samples. Significantly, combining miRs 26b-5p and 98-5p, we developed a model that has the best predictive accuracy (94%) in discriminating prostate cancer from non-cancer. Combining miR-26b-5p and miR-4732-3p, we have a good diagnostic accuracy (80%) for high-grade prostate cancer patients. Our results for high-grade prostate cancer could be partially related to small numbers of patients enrolled in our trial but at this stage they still represent the same accuracy for the detection of clinically significant prostate cancer as suggested by the current series on MRI [6]. If our results are confirmed in the ongoing multicenter trial, these new biomarkers could be used as adjunctive markers for prostate cancer diagnosis and importantly, to spare unnecessary biopsy. These new biomarkers’ possible role in patients with a PIRADS 3 on MRI is also under evaluation and could further increase our findings’ clinical application. Our results represent an accurate option to identify patients at risk of prostate cancer and particularly of clinically significant cancer in patients with no pre-biopsy MRI.

## Conclusions

We developed and evaluated two different two mir-based tests’ clinical feasibility to diagnose prostate cancer, respectively. The finding suggests that both tests could be promising biomarkers in patients with PCa.

## Supplementary Information

**Additional file 1: Figure S1.** HeatMap.

**Additional file 2: Table S1.** Cluster analysis identified two main cluster.

**Additional file 3: Figure S2.** Calibration plots.

**Additional file 4: Figure S3.** Differential levels of miRs and PSA in grades group.

**Additional file 5: Figure S4.** ROC curve analysis of selected miRs and PSA in high-grade tumors.

**Additional file 6: Table S2.** MiRs model.

**Additional file 7: Figure S5.** Calibration plots.

## Data Availability

Raw data are available in GEO with accession number GSE118038.

## Abbreviations

PCa: Prostate cancer
PSA: Prostate-specific antigen
DRE: Digital rectal examination
BPH: Benign prostate hyperplasia
dDPCR: Droplet Digital PCR
TRUS: Transrectal ultrasound
ROC: Receiver operator curve
AUC: Area under the curve
CI: Confidence interval
