# Supplementary figures and images for: A preliminary study of micro-RNAs as minimally invasive biomarkers for the diagnosis of prostate cancer patients
Source: J Exp Clin Cancer Res. 2021 Feb 23;40:79. doi: 10.1186/s13046-021-01875-0 (PMC7903618; doi:10.1186/s13046-021-01875-0)

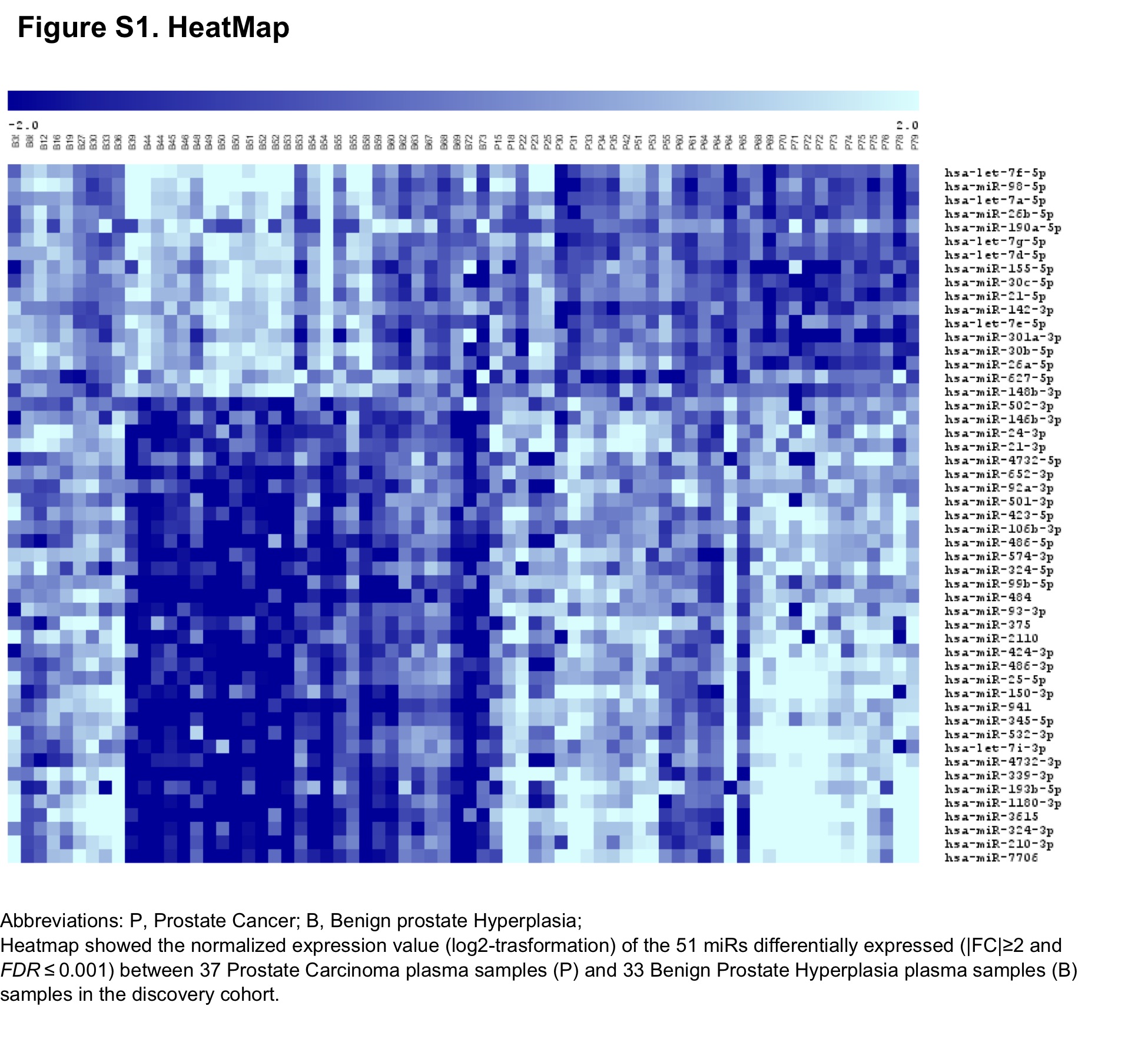

Supplement: Supplementary file 1 — Additional file 1: Figure S1. HeatMap. [file 13046_2021_1875_MOESM1_ESM.jpg]

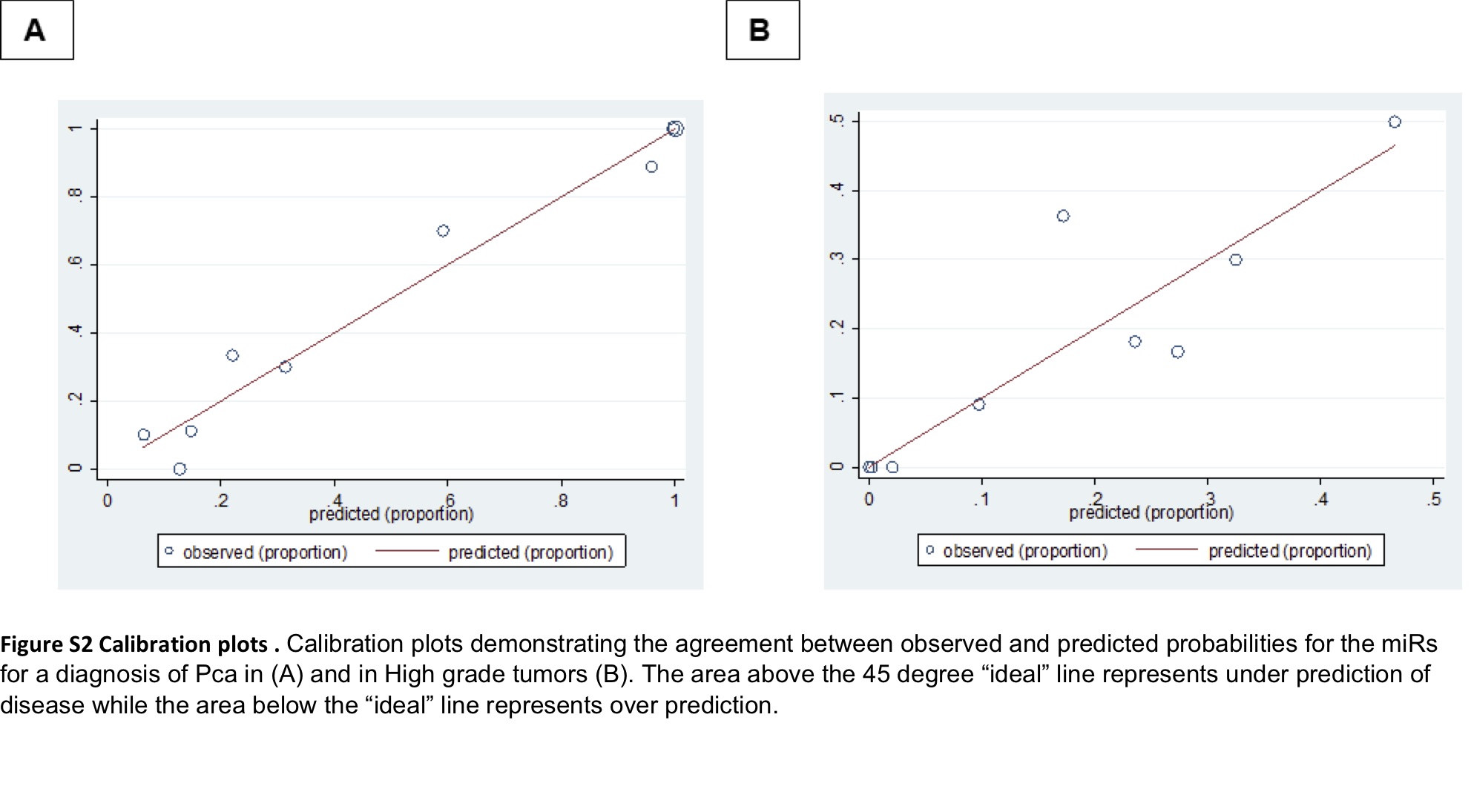

Supplement: Supplementary file 3 — Additional file 3: Figure S2. Calibration plots. [file 13046_2021_1875_MOESM3_ESM.jpg]

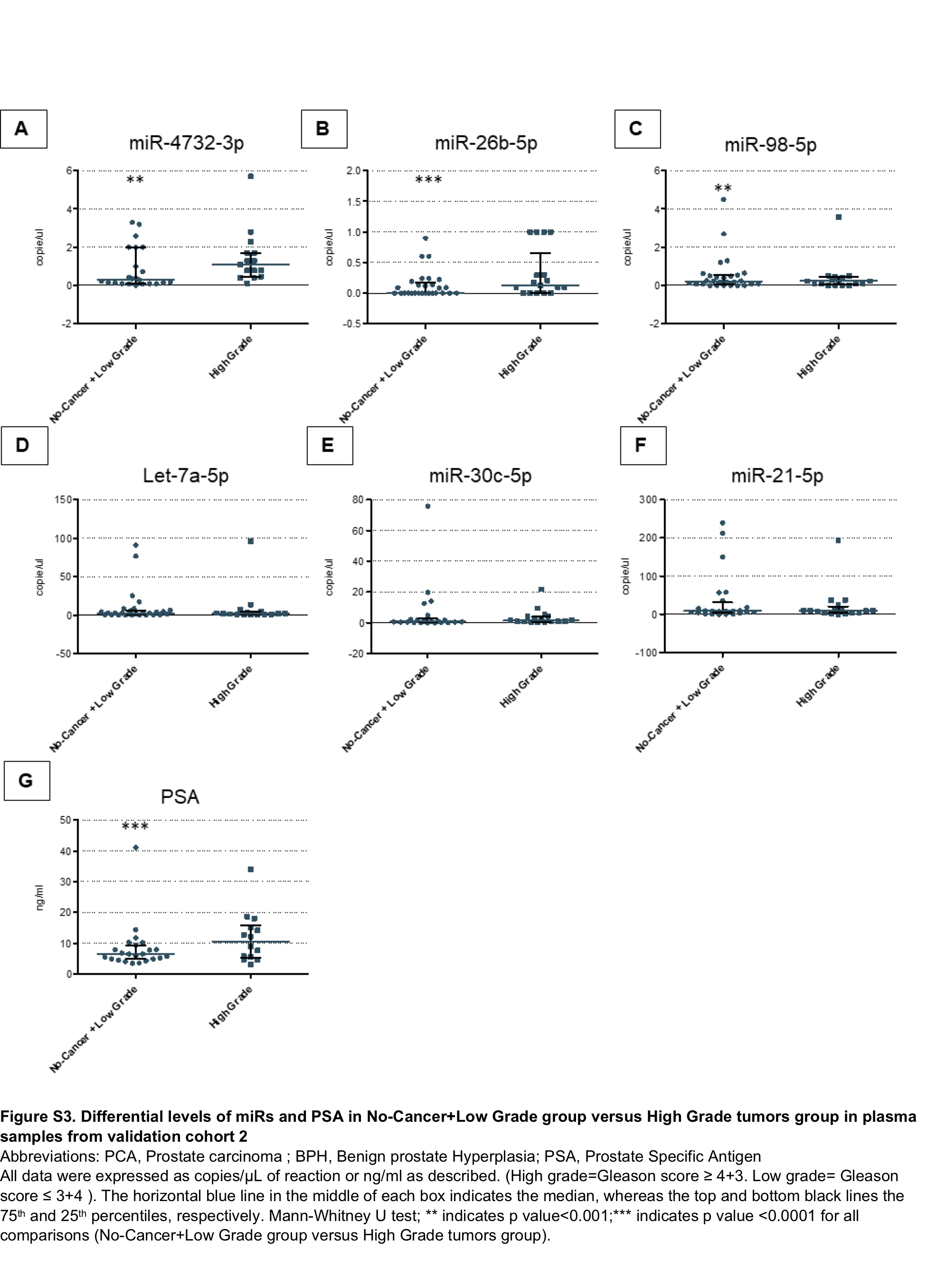

Supplement: Supplementary file 4 — Additional file 4: Figure S3. Differential levels of miRs and PSA in grades group. [file 13046_2021_1875_MOESM4_ESM.jpg]

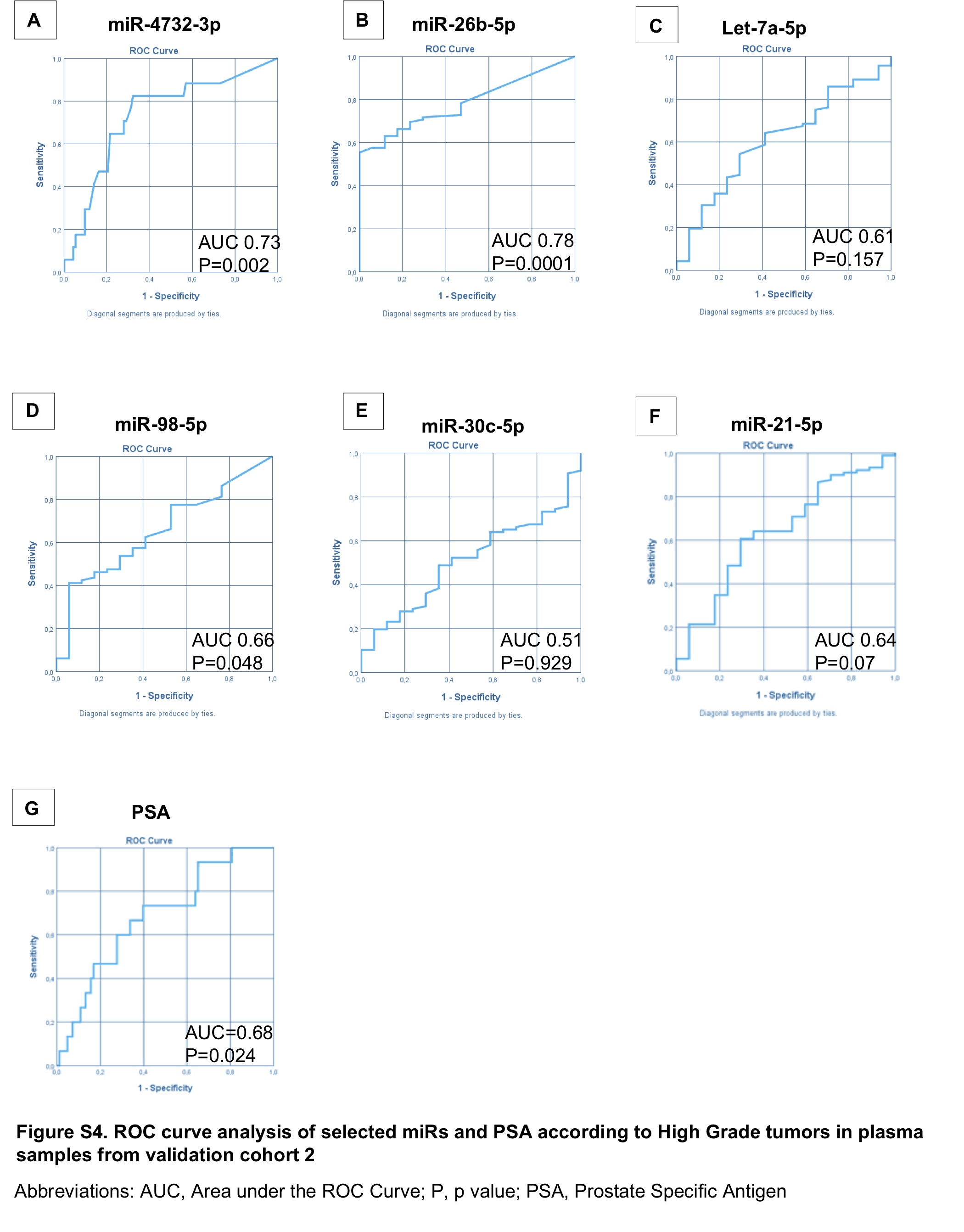

Supplement: Supplementary file 5 — Additional file 5: Figure S4. ROC curve analysis of selected miRs and PSA in high-grade tumors. [file 13046_2021_1875_MOESM5_ESM.jpg]

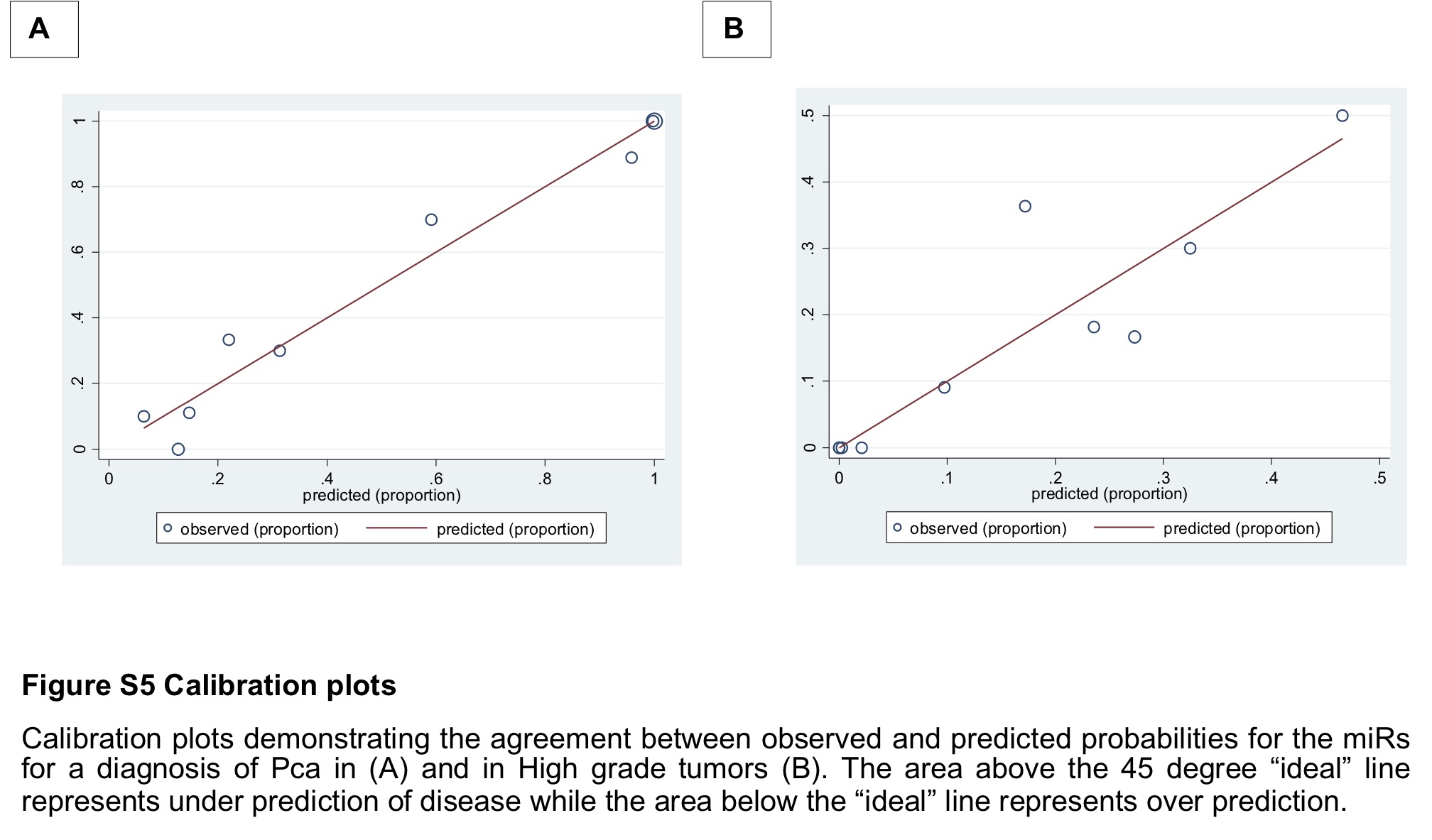

Supplement: Supplementary file 7 — Additional file 7: Figure S5. Calibration plots. [file 13046_2021_1875_MOESM7_ESM.jpg]
