# Supplementary material for: A preliminary study of micro-RNAs as minimally invasive biomarkers for the diagnosis of prostate cancer patients
Source: J Exp Clin Cancer Res. 2021 Feb 23;40:79. doi: 10.1186/s13046-021-01875-0 (PMC7903618; doi:10.1186/s13046-021-01875-0)
Supplement: Supplementary file 2 — Additional file 2: Table S1. Cluster analysis identified two main cluster. [file 13046_2021_1875_MOESM2_ESM.docx]

**Table S1. Cluster analysis identified two main cluster**

| **SAMPLE** | **DIAGNOSIS** | **CLUSTER** | **SAMPLE** | **DIAGNOSIS** | **CLUSTER** |
| --- | --- | --- | --- | --- | --- |
| B35 | BPH | 1 | B88 | BPH | 2 |
| B276 | BPH | 1 | B129 | BPH | 2 |
| B303 | BPH | 1 | B167 | BPH | 2 |
| B337 | BPH | 1 | B190 | BPH | 2 |
| B385 | BPH | 1 | B391 | BPH | 2 |
| P189 | PCa | 1 | B444 | BPH | 2 |
| P223 | PCa | 1 | B449 | BPH | 2 |
| P308 | PCa | 1 | B452 | BPH | 2 |
| P319 | PCa | 1 | B465 | BPH | 2 |
| P336 | PCa | 1 | B489 | BPH | 2 |
| P346 | PCa | 1 | B491 | BPH | 2 |
| P350 | PCa | 1 | B504 | BPH | 2 |
| P534 | PCa | 1 | B507 | BPH | 2 |
| P649 | PCa | 1 | B517 | BPH | 2 |
| P686 | PCa | 1 | B520 | BPH | 2 |
| P695 | PCa | 1 | B524 | BPH | 2 |
| P700 | PCa | 1 | B531 | BPH | 2 |
| P717 | PCa | 1 | B536 | BPH | 2 |
| P721 | PCa | 1 | B540 | BPH | 2 |
| P724 | PCa | 1 | B546 | BPH | 2 |
| P734 | PCa | 1 | B551 | BPH | 2 |
| P747 | PCa | 1 | B554 | BPH | 2 |
| P755 | PCa | 1 | B587 | BPH | 2 |
| P758 | PCa | 1 | B597 | BPH | 2 |
| P766 | PCa | 1 | B606 | BPH | 2 |
| P781 | PCa | 1 | B627 | BPH | 2 |
| P790 | PCa | 1 | B638 | BPH | 2 |
|  |  |  | B676 | BPH | 2 |
|  |  |  | B680 | BPH | 2 |
|  |  |  | B694 | BPH | 2 |
|  |  |  | B727 | BPH | 2 |
|  |  |  | B737 | BPH | 2 |
|  |  |  | P158 | PCa | 2 |
|  |  |  | P233 | PCa | 2 |
|  |  |  | P254 | PCa | 2 |
|  |  |  | P426 | PCa | 2 |
|  |  |  | P557 | PCa | 2 |
|  |  |  | P607 | PCa | 2 |
|  |  |  | P618 | PCa | 2 |
|  |  |  | P640 | PCa | 2 |
|  |  |  | P642 | PCa | 2 |
|  |  |  | P655 | PCa | 2 |

Abbreviations: PCa, Prostate carcinoma ; BPH, Benign prostate Hyperplasia.
