# Supplementary material for: A preliminary study of micro-RNAs as minimally invasive biomarkers for the diagnosis of prostate cancer patients
Source: J Exp Clin Cancer Res. 2021 Feb 23;40:79. doi: 10.1186/s13046-021-01875-0 (PMC7903618; doi:10.1186/s13046-021-01875-0)
Supplement: Supplementary file 6 — Additional file 6: Table S2. MiRs model. [file 13046_2021_1875_MOESM6_ESM.docx]

**Table S2. MiRs model**

| **miRs** | **Cancer Status Model** | | **miRs** | **Tumor Grade Status Model** | |
| --- | --- | --- | --- | --- | --- |
|  | AUC (CI) | P Value |  | AUC (CI) | P Value |
| miR-26b-5p  miR-98-5p | 0.944(0.835-0.954) | **.001** | miR-26b-5p  miR-4732-3p | 0.80(0.69-0.873) | **.0001** |

Abbreviations: AUC, Area under the curve: CI, Confidence Interval.
